# Supplementary material for: KOSMOS: An Open Source Underwater Video Lander for Monitoring Coastal Fishes and Habitats
Source: Sensors (Basel). 2021 Nov 20;21(22):7724. doi: 10.3390/s21227724 (PMC8619907; doi:10.3390/s21227724)
Supplement: Supplementary file 1 [file sensors-21-07724-s001.zip › sensors-1442412-supplementary.pdf]

Table S1. Screenshots from the KOSMOS, STAVIRO-Sony and STAVIRO-Paralenz systems at distances ranging between 1 and 6 m.

KOSMOS	STAVIRO	Paralenz
1.1 m	1.05 m	1.07 m
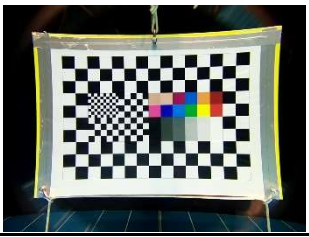	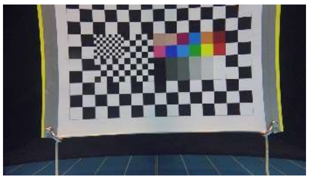	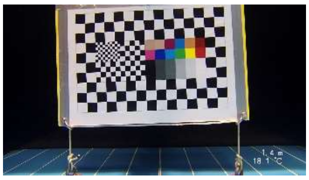
2.01 m	1.99 m	2.07 m
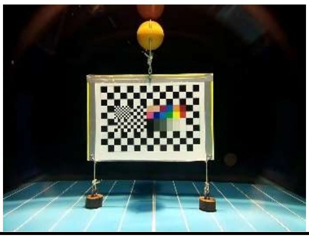	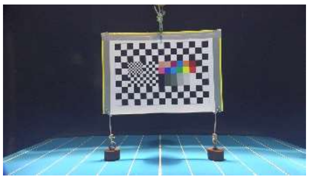	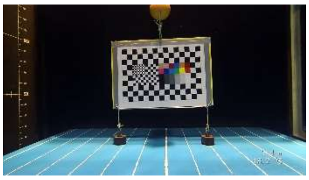
2.98 m	3.05 m	3.02 m
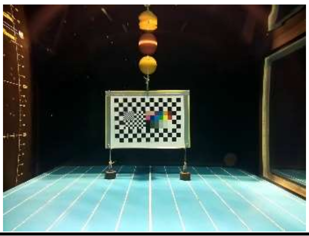	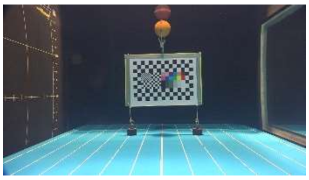	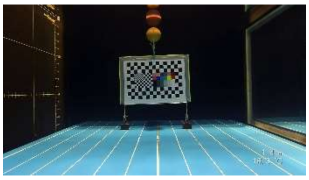
3.98 m	3.99 m	4.04 m
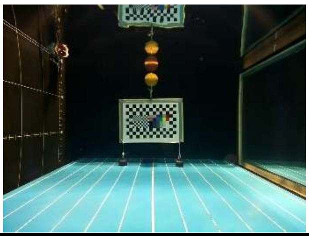	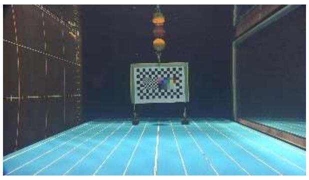	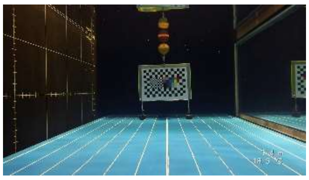
5.06 m	4.98 m	5.04 m
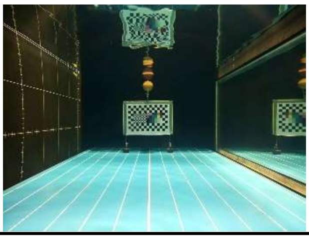	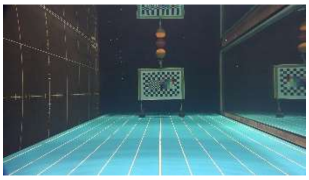	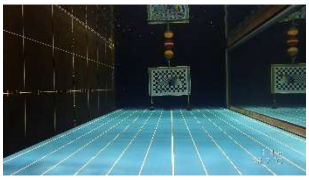
6.07 m	5.98 m	6.02 m
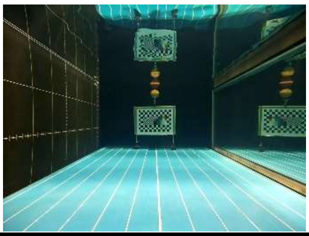	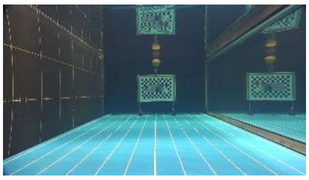	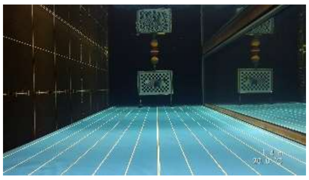

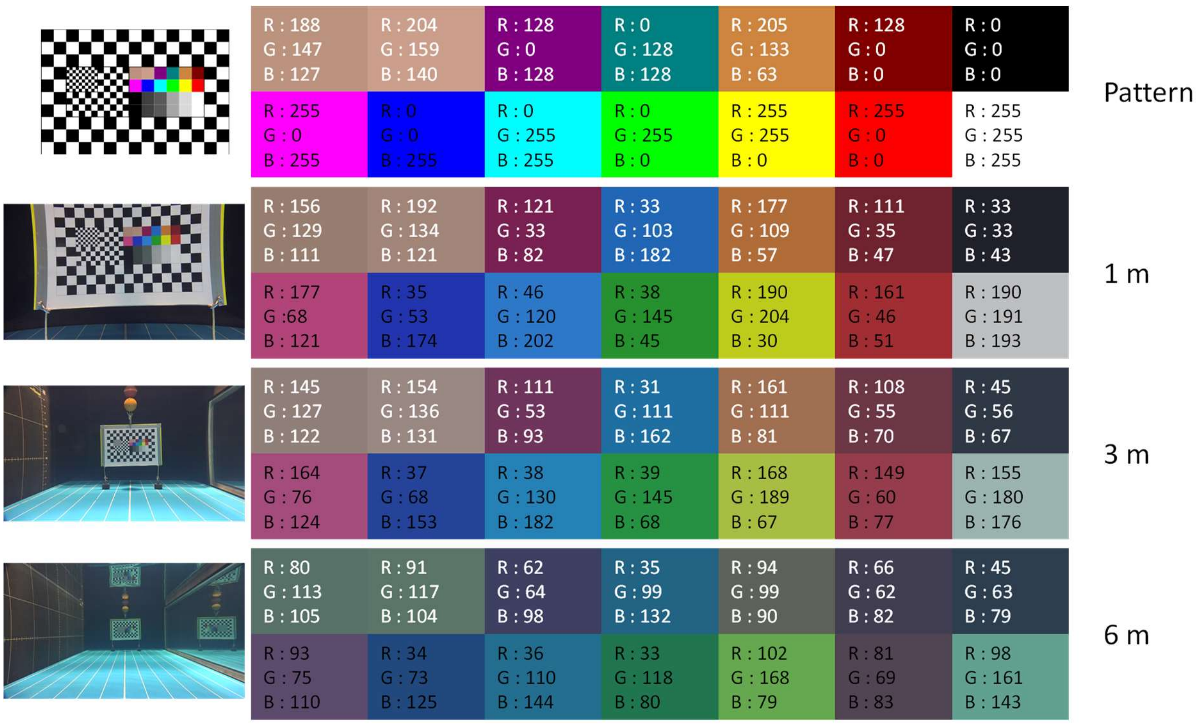

Pattern	R : 188 G : 147 B : 127	R : 204 G : 159 B : 140	R : 128 G : 0 B : 128	R : 0 G : 128 B : 128	R : 205 G : 133 B : 63	R : 128 G : 0 B : 0	R : 0 G : 0 B : 0
	R : 255 G : 0 B : 255	R : 0 G : 0 B : 255	R : 0 G : 255 B : 255	R : 0 G : 255 B : 0	R : 255 G : 255 B : 0	R : 255 G : 0 B : 0	R : 255 G : 255 B : 255
1 m	R : 156 G : 129 B : 111	R : 192 G : 134 B : 121	R : 121 G : 33 B : 82	R : 33 G : 103 B : 182	R : 177 G : 109 B : 57	R : 111 G : 35 B : 47	R : 33 G : 33 B : 43
	R : 177 G :68 B : 121	R : 35 G : 53 B : 174	R : 46 G : 120 B : 202	R : 38 G : 145 B : 45	R : 190 G : 204 B : 30	R : 161 G : 46 B : 51	R : 190 G : 191 B : 193
3 m	R : 145 G : 127 B : 122	R : 154 G : 136 B : 131	R : 111 G : 53 B : 93	R : 31 G : 111 B : 162	R : 161 G : 111 B : 81	R : 108 G : 55 B : 70	R : 45 G : 56 B : 67
	R : 164 G : 76 B : 124	R : 37 G : 68 B : 153	R : 38 G : 130 B : 182	R : 39 G : 145 B : 68	R : 168 G : 189 B : 67	R : 149 G : 60 B : 77	R : 155 G : 180 B : 176
6 m	R : 80 G : 113 B : 105	R : 91 G : 117 B : 104	R : 62 G : 64 B : 98	R : 35 G : 99 B : 132	R : 94 G : 99 B : 90	R : 66 G : 62 B : 82	R : 45 G : 63 B : 79
	R : 93 G : 75 B : 110	R : 34 G : 73 B : 125	R : 36 G : 110 B : 144	R : 33 G : 118 B : 80	R : 102 G : 168 B : 79	R : 81 G : 69 B : 83	R : 98 G : 161 B : 143

Figure S1. STAVIRO-Sony RGB coefficients at distances of 1, 3 and 6 m. Top: RGB coefficients for test pattern.

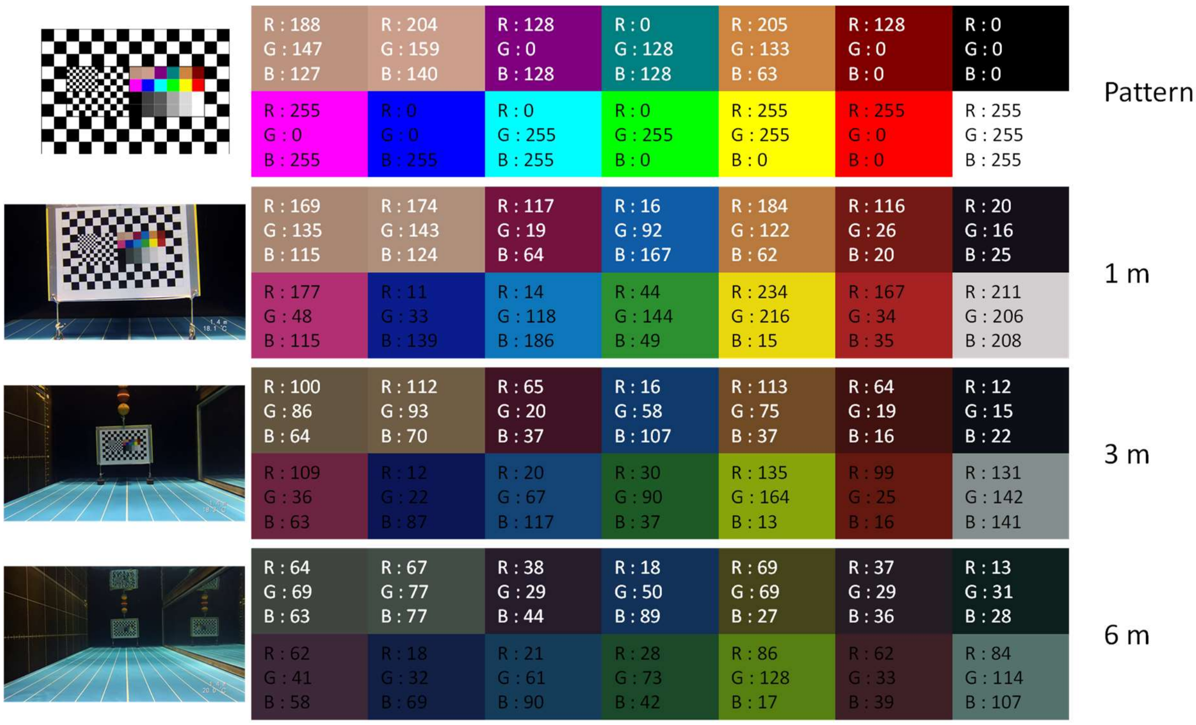

Pattern	R : 188 G : 147 B : 127	R : 204 G : 159 B : 140	R : 128 G : 0 B : 128	R : 0 G : 128 B : 128	R : 205 G : 133 B : 63	R : 128 G : 0 B : 0	R : 0 G : 0 B : 0
	R : 255 G : 0 B : 255	R : 0 G : 0 B : 255	R : 0 G : 255 B : 255	R : 0 G : 255 B : 0	R : 255 G : 255 B : 0	R : 255 G : 0 B : 0	R : 255 G : 255 B : 255
1 m	R : 169 G : 135 B : 115	R : 174 G : 143 B : 124	R : 117 G : 19 B : 64	R : 16 G : 92 B : 167	R : 184 G : 122 B : 62	R : 116 G : 26 B : 20	R : 20 G : 16 B : 25
	R : 177 G : 48 B : 115	R : 11 G : 33 B : 139	R : 14 G : 118 B : 186	R : 44 G : 144 B : 49	R : 234 G : 216 B : 15	R : 167 G : 34 B : 35	R : 211 G : 206 B : 208
3 m	R : 100 G : 86 B : 64	R : 112 G : 93 B : 70	R : 65 G : 20 B : 37	R : 16 G : 58 B : 107	R : 113 G : 75 B : 37	R : 64 G : 19 B : 16	R : 12 G : 15 B : 22
	R : 109 G : 36 B : 63	R : 12 G : 22 B : 87	R : 20 G : 67 B : 117	R : 30 G : 90 B : 37	R : 135 G : 164 B : 13	R : 99 G : 25 B : 16	R : 131 G : 142 B : 141
6 m	R : 64 G : 69 B : 63	R : 67 G : 77 B : 77	R : 38 G : 29 B : 44	R : 18 G : 50 B : 89	R : 69 G : 69 B : 27	R : 37 G : 29 B : 36	R : 13 G : 31 B : 28
	R : 62 G : 41 B : 58	R : 18 G : 32 B : 69	R : 21 G : 61 B : 90	R : 28 G : 73 B : 42	R : 86 G : 128 B : 17	R : 62 G : 33 B : 39	R : 84 G : 114 B : 107

Figure S2. STAVIRO-Paralenz RGB coefficients at distances of 1, 3 and 6 m. Top: RGB coefficients for test pattern.
